# Supplementary material for: Low N2O and variable CH4 fluxes from tropical forest soils of the Congo Basin
Source: Nat Commun. 2022 Jan 17;13:330. doi: 10.1038/s41467-022-27978-6 (PMC8764088; doi:10.1038/s41467-022-27978-6)
Supplement: Supplementary file 1 — Supplementary Information [file 41467_2022_27978_MOESM1_ESM.pdf]

Low N_2O and variable CH_4 fluxes from tropical forest soils of the Congo Basin

Supplementary Table 1 | General site information, including sampling duration and periodicity of different datasets used. Core long-term observation sites are highlighted in bold. Terrestrial and aquatic sites are marked in grey and blue respectively.

LOCATION<br>Latitude, longitude, elevation	FOREST/<br>SOIL TYPE	DATES	PERIODICITY	REP	*n*	*n* isotopes
**Kahuzi-Biéga National Park**	**Montane**	06.09.2016 - 19.09.2016[§]	1 d	7		
S 2.31314, E 28.75471, 2050 m	Umbric Ferralsol	26.04.2017 - 02.05.2017[§]	1 d	3	**398**	**12**
		06.05.2017 - 24.03.2018	7 d	3		
		29.03.2018 - 26.03.2019*[~]	14 d	5		
**Jardin Botanique d'Eala**	**Swamp**	19.11.2019 - 25.11.2019	1 d	5	**266**	**6**
N 0.06335, E 18.31054, 300 m	Eutric Gleysol	26.11.2019 - 07.12.2020[~]	14 d	6		
**Yoko Forest Reserve**	**Lowland**	26.09.2016 - 09.10.2016[§]	1 d	7		
N 0.29299, E 25.30111, 486 m	Xanthic Ferralsol	05.05.2017 - 01.06.2017[§]	1 d	6		
		31.10.2016 - 18.12.2017	7 d	6	**754**	**32**
		17.03.2018 - 30.03.2019*[~]	14 d	5		
		22.04.2019 - 01.03.2020	14 d	5		
Yangambi Biosphere Reserve	Lowland	11.10.2016 - 24.10.2016	1 d	7	98	7
N 0.81128, E 24.49041, 480 m	Xanthic Ferralsol					
Maringa-Lopori-Wamba Landscape	Lowland	27.04.2016 - 01.08.2016	irregular	3	42	6
N 0.67208, E 22.33583, 386 m	Xanthic Ferralsol					
TOTAL TERRESTRIAL MEASUREMENTS					1558	63
**Langa River**	**Montane**	29.03.2018 - 26.03.2019	14 d	3	**80**	**14**
S 2.30674, E 28.75554	Umbric Ferralsol					
**Yoko River**	**Lowland**	17.03.2018 - 30.03.2019	14 d	3	**78**	**9**
N 0.29320, E 25.29481	Xanthic Ferralsol					
**Lolifa River**	**Swamp**	26.11.2019 - 07.12.2020[~]	14 d	3	**84**	
S 0.03135, E 18.3102	Eutric Gleysol					
Buba River	Montane	29.03.2018, 27.08.2018		5	10	
S 2.33138, E 28.75651	Umbric Ferralsol					
Cirado River	Montane	29.03.2018, 27.08.2018		5	10	
S 2.333778, E 28.75445	Umbric Ferralsol					
Isalowe River	Lowland	13.03.2018, 19.09.2018		5	10	
N 0.79808, E 24.50101	Xanthic Ferralsol					
Boonde River	Lowland	13.03.2018, 19.09.2018		5	10	
N 0.77434, E 24.39577	Xanthic Ferralsol					
Lobilo River	Lowland	15.03.2018, 19.09.2018		5	10	
N 0.76081, E 24.59414	Xanthic Ferralsol					
Lokombe River	Lowland	15.03.2018, 19.09.2018		5	10	
N 0.71624, E 24.605	Xanthic Ferralsol					
Bekele River	Lowland	15.03.2018, 19.09.2018		5	10	
N 0.69606, E 24.60126	Xanthic Ferralsol					
Losa River	Lowland	15.03.2018, 19.09.2018		5	10	
N 0.6587, E 24.64186	Xanthic Ferralsol					
Lusambila River	Lowland	13.03.2018, 19.09.2018		5	10	
N 0.75728, E 24.48266	Xanthic Ferralsol					
TOTAL AQUATIC MEASUREMENTS					332	23

[§]N_2O dataset partly published in Bauters et al. 2019; *N_2O dataset published in Gallarotti et al. 2021. [~]Period during which complementing riverine samples were taken from headwater streams draining the same catchments in which core sites were located.

Supplementary Table 2 | Summary statistics. All numbers are given in nmol m^{-2} s^{-1} except last line.

		Lowland	Montane	Swamp non-inundated	Swamp inundated
CH_4	Geometric mean (logdistributed density of the fluxes)	**-0.93**	**-1.13**	**0.71**	**90.05**
	Arithmetic mean*	-0.85	-1.19	38.76	445.99
	low 95% confidence interval	-1.13	-1.24	-0.80	38.88
	high 95% confidence interval	-0.71	-1.01	3.20	210.94
	variability in fluxes explained by soil temperature and WFPS (marginal R^2)	0.00	0.33	0.26	no data
N_2O	Geometric mean (logdistributed density of the fluxes)	**0.18**	**0.11**	**0.40**	**-0.02**
	Arithmetic mean*	0.14	0.18	1.84	0.14
	low 95% confidence interval	0.14	0.07	0.21	-0.09
	high 95% confidence interval	0.22	0.15	0.72	0.09
	variability in fluxes explained by soil temperature and WFPS (marginal R^2)	0.05	0.27	0.29	no data

*Overall arithmetic mean via 1) arithmetic mean over chambers per site and per sampling date, and subsequently 2) arithmetic mean of this mean over dates

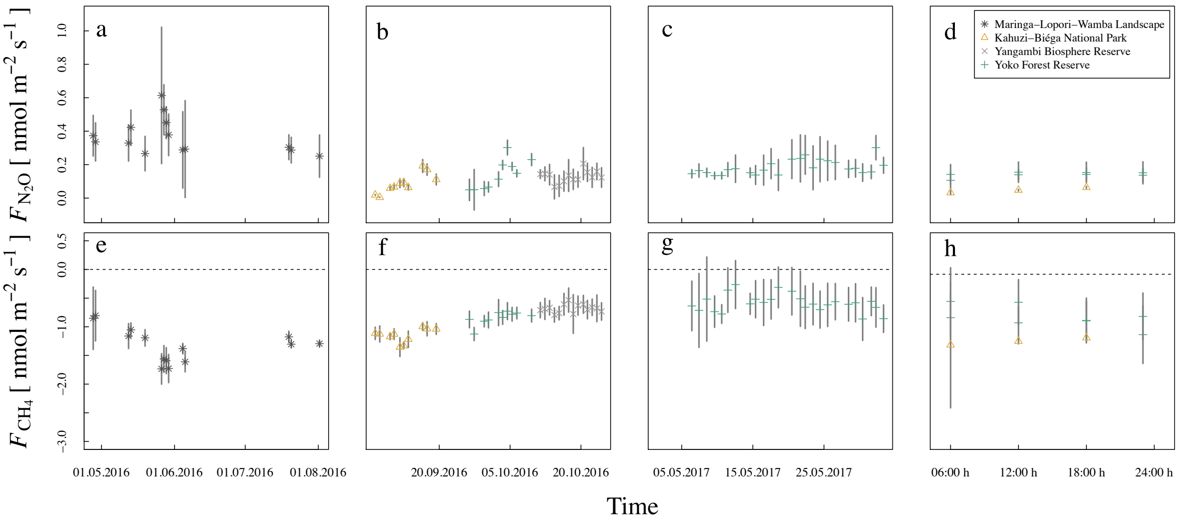

Supplementary Figure 1 | Soil N_2O fluxes measured during the initial short-term campaigns in 2016 and 2017 (see Table 1) at **a** monthly (Maringa-Lopori-Wamba Landscape) **b, c** daily (Yoko Forest Reserve, Yangambi Biosphere Reserve, Kahuzi-Biéga National Park) to **d** sub-daily resolution (Yoko Forest Reserve, Kahuzi-Biéga National Park) to determine spatiotemporal variation. Error bars indicate standard error. **e, f, g and h** are corresponding panels for CH_4 fluxes.

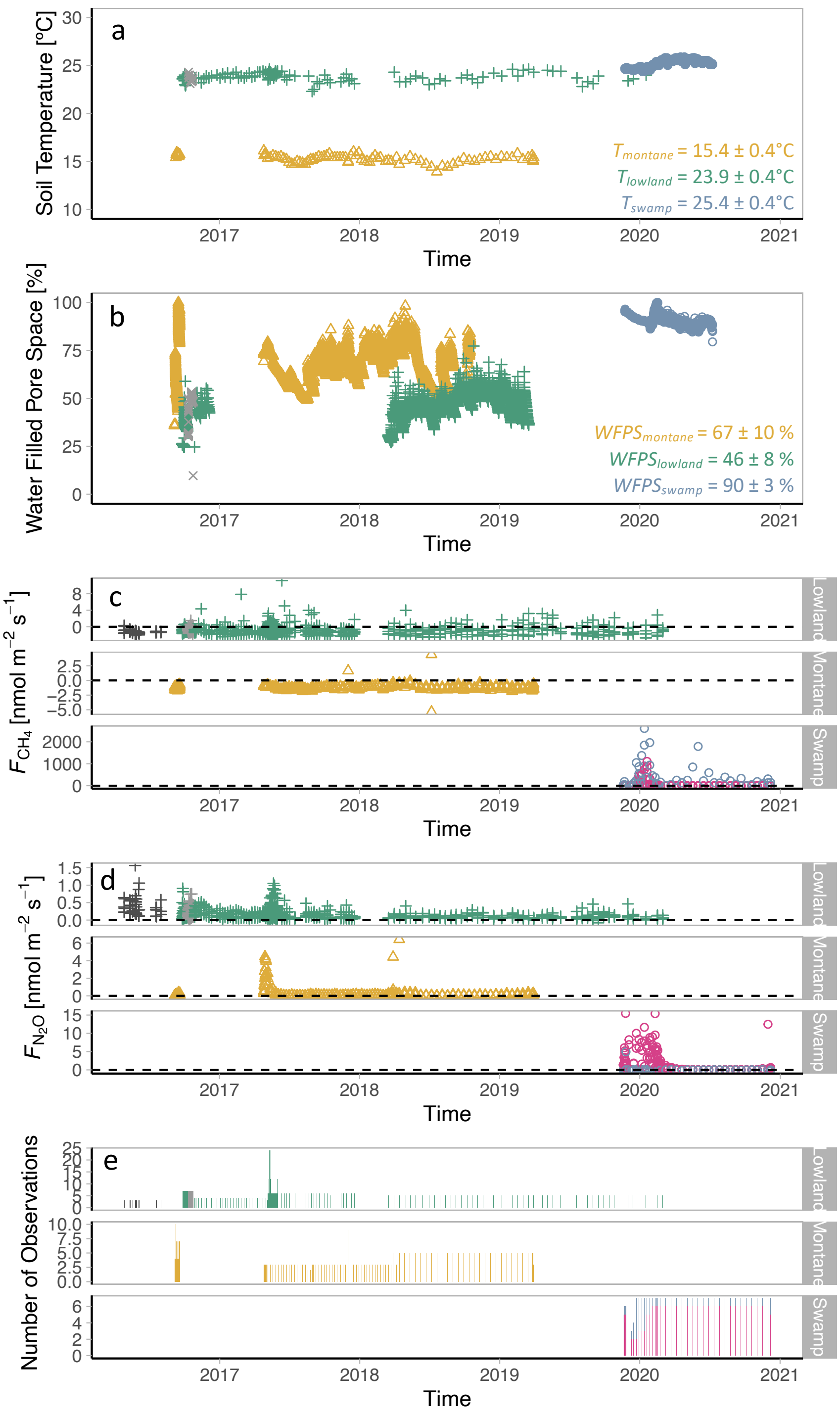

Supplementary Figure 2 | a Soil temperature at 30 cm depth **b** water filled pore space measurements at 30 cm soil depth **c** CH_4 fluxes separated by forest type **d** N_2O fluxes separated by forest type **e** number of daily observations separated by forest type. Darkgrey – Maringa-Lopori-Wamba Landscape; Lightgrey – Yangambi Biosphere Reserve; Green – Yoko Forest Reserve; Yellow – Kahuzi-Biéga National Park; Blue – Swamp Forest inundated (Jardin Botanique d'Eala); Pink – Swamp Forest non-inundated (Jardin Botanique d'Eala)

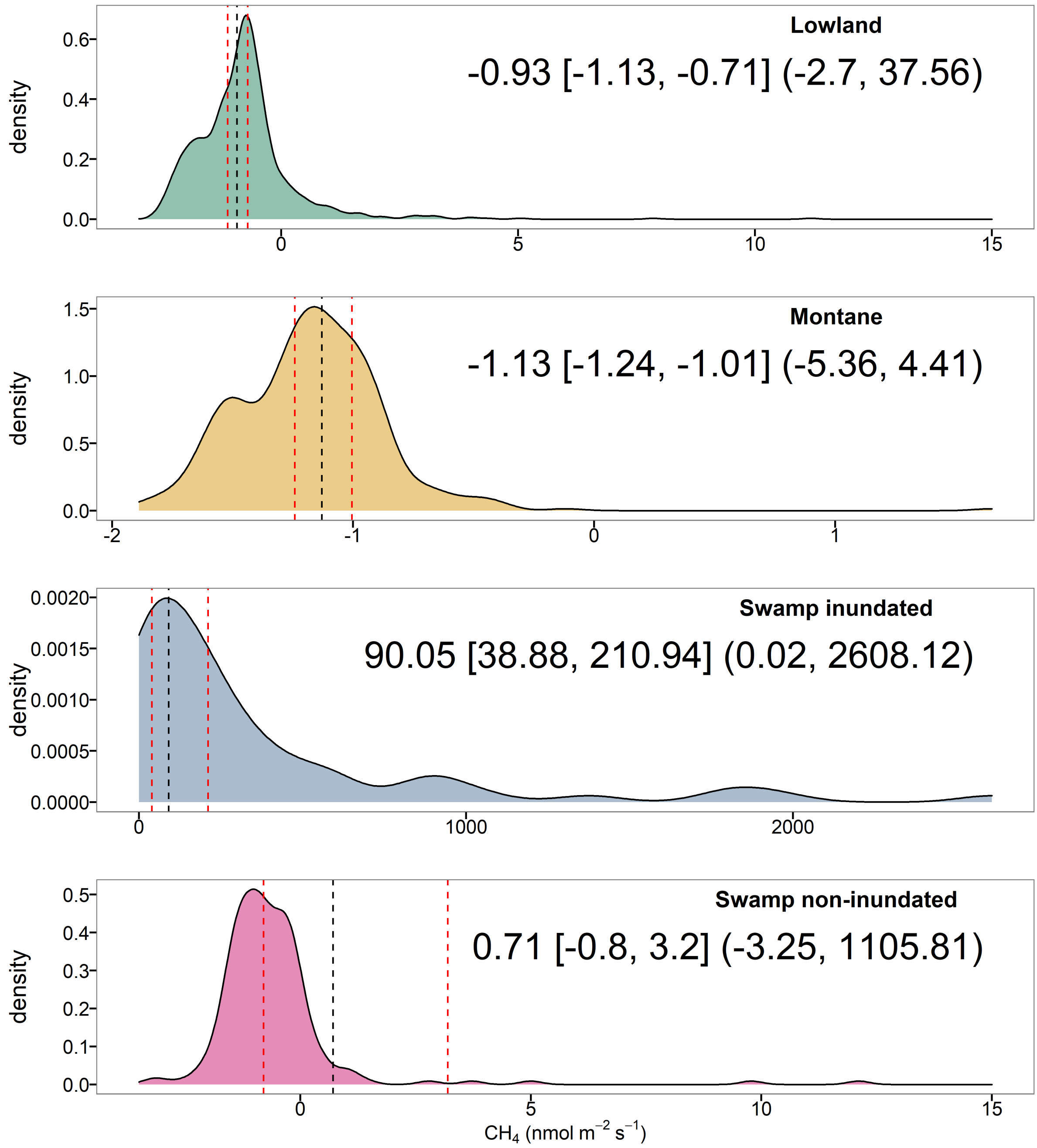

Supplementary Figure 3 | Density distribution functions of CH_4 fluxes for all studied forest types. Black dashed line indicates geometric mean and red dashed lines lower and upper 95% confidence intervals (CI). Number insets are geometric mean [lower CI, upper CI](min, max) in nmol m^{-2} s^{-1}.

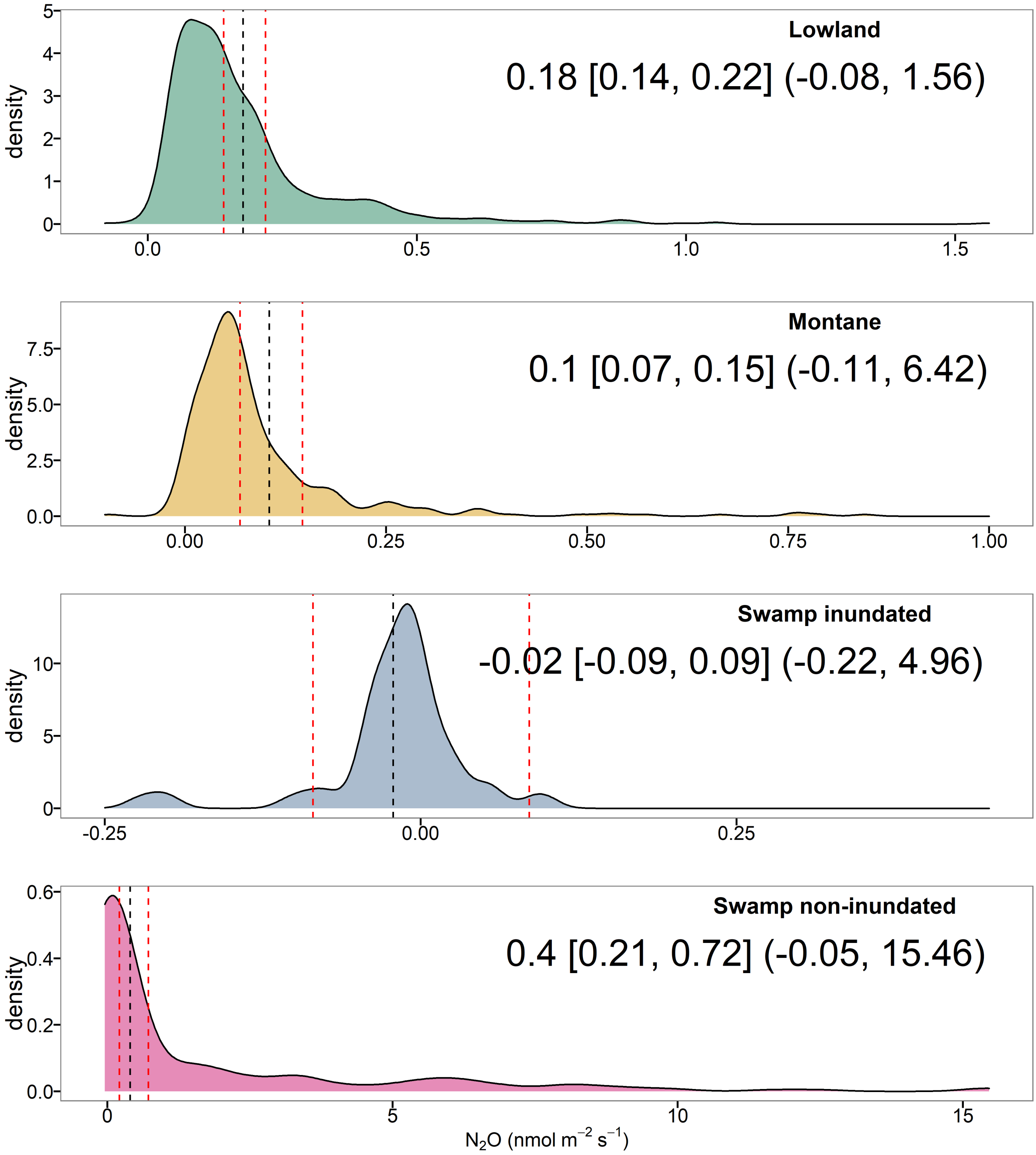

Supplementary Figure 4 | Density distribution functions of N_2O fluxes for all studied forest types. Black dashed line indicates geometric mean and red dashed lines lower and upper 95% confidence intervals (CI). Number insets are geometric mean [lower CI, upper CI](min, max) in nmol m^{-2} s^{-1}.

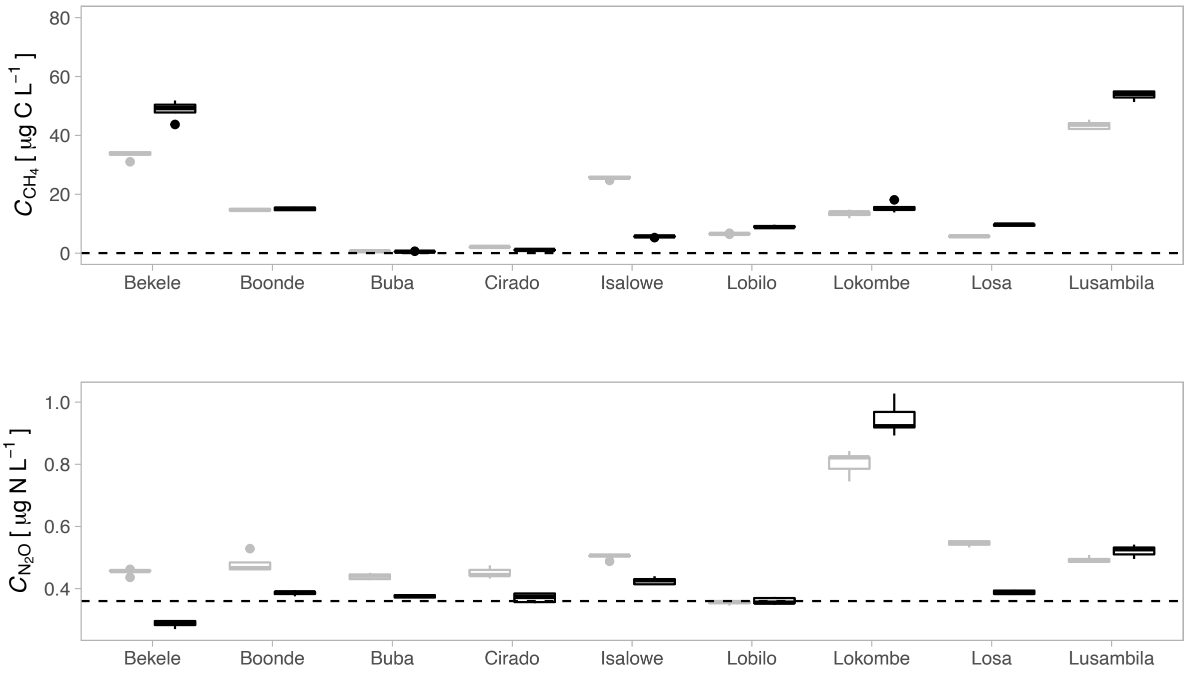

Supplementary Figure 5 | Spatial variation of dissolved CH_4 and N_2O in stream water at different pristine forest catchments in geographical vicinity of the core site catchments (see Fig S6). Note, all streams drain lowland forest catchments apart from site Buba and Cirado which drain montane forest catchments (for more information see Table S1). Black indicates wet season and grey dry season. Black dashed line indicates N_2O (0.36 µg N L^{-1}) and CH_4 (0.03 µg C L^{-1}) at equilibrium with the atmosphere.

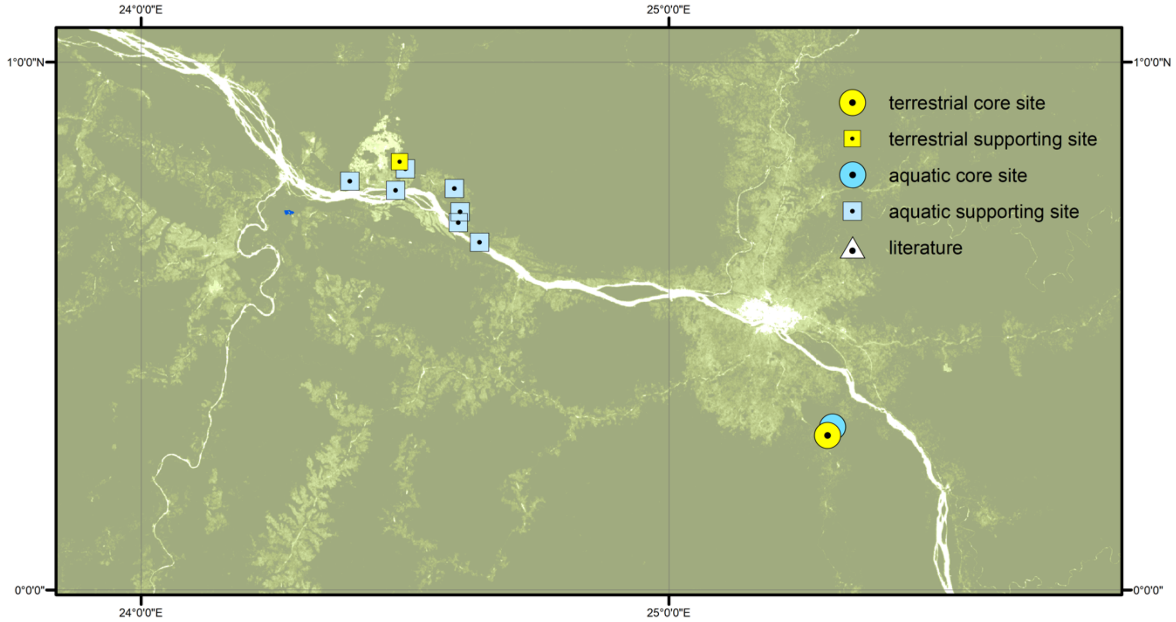

Supplementary Figure 6 | Zoom-in version of Figure 1 with locations of supporting riverine sampling sites draining into the Congo River (blue squares), all situated relatively close to the Yangambi Biosphere Reserve (yellow square). Blue and yellow circle represents Yoko Forests Reserve core site.

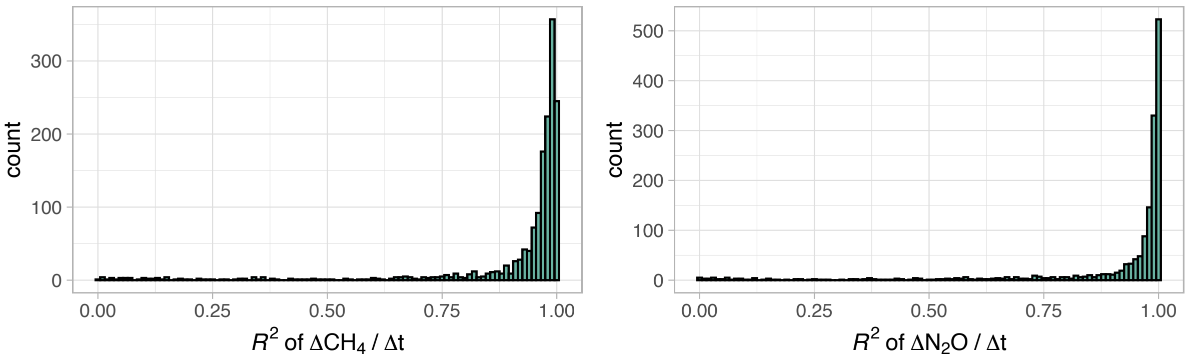

Supplementary Figure 7 | Coefficient of determination (R^2) histograms of each individual regression of CH_4 and N_2O concentrations vs. time, respectively.

Deriving dissolved gas concentrations using the headspace equilibration technique

The following supplemental file serves to reproduce calculations for dissolved concentrations of N_2O and CH_4 using the headspace equilibration technique based on the SOP by Bastviken (2015). In the field, 6mL of bubble free water was injected into a N_2 flushed 12mL vial (Labco exetainer, UK) containing 50 μL of 50% (w/v) $ZnCl_2$ solution (Dong *et al.*, 2006)[1]. The following protocol requires to convert the mole fraction of a gas x measured by a gas chromatograph [C_{ppm}typically in ppm, i.e. μmol mol^{-1}] to the partial pressure of that gas x in $atm \cdot mol \cdot mol^{-1}$:

$$P_x = \frac{C_{ppm}}{10^6} \cdot P_{headspace},$$

with $P_{headspace}$ as the total pressure of the gas mixture in the vial[2] (gas phase under equilibrium conditions) given in atm. This is equivalent to the headspace pressure, and can be calculated as follows:

$$P_{headspace} = P_{lab} \cdot \frac{V_{vial}}{(V_{vial} - V_{aq})}$$

, where V_{aq} denotes the total water volume injected into the exetainer [in L], V_{vial} the total vial volume [in L] and P_{lab} the laboratory pressure at time of flushing [in atm]. The total dissolved gas concentration (C_{total}) using the headspace equilibration technique is the sum of the partial gas pressure which partitioned into the gas phase (C_h) and the partial gas pressure remaining in the aqueous phase (C_{aq}):

$$C_{total} = \frac{C_h + C_{aq}}{V_{aq}}$$

C_h is derived via

$$C_h = \frac{P_x \cdot V_{hs}}{R \cdot T}$$

, where P_x is the partial gas pressure in the headspace derived from initial gas chromatography measurements in $atm \cdot mol \cdot mol^{-1}$, V_{hs} the headspace volume in L, R the universal gas constant in L atm mol^{-1} K^{-1}, and T the laboratory temperature in K (where equilibration took place). The gas concentration still dissolved in the aqueous phase C_{aq} is derived via

$$C_{aq} = P_x \cdot V_{aq} \cdot K_H$$

, where K_H denotes Henry's constant for the respective trace gas.

The **Henry's law constant** is either defining the volatility of a gas species from solution to the atmosphere, or the solubility of a given gas from atmosphere to solution. In the latter definition, the constants' value is increasing with increasing solubility and is species specific. Henry's law solubility constant is defined as:

$$K_H = \frac{C_{aq}}{P_x}$$

, with K_H in units of $\frac{mol}{kg \cdot bar}$ or $\frac{mol}{L \cdot atm}$ [3]. The K_H coefficients used in this SOP are taken from the NIST Standard Reference Database:

$K_{H|CH_4}$ = 0.0014
$K_{H|N_2O}$ = 0.025

at 298.15 K.

Example R code to determine dissolved gas concentrations of nitrous oxide. Note, for methane the respective Henrys constant as well as molecular weight need to be changed accordingly.

```
# ######################
# global parameters ####
# ######################

K                 = 0.025    # HENRY's law constant for N2O in mol L-1 atm-
1

V.vial            = 0.012          # volume vial in L
V.aq              = 0.006          # volume of water in L
V.hs              = V.vial - V.aq  # volume gas in headspace in L

R                 = 0.08206       # universal gas constant in L atm mol-1 K
-1
T                 = 273.15 + 24   # laboratory temperature in K; ETHZ GC
Laboratory 24 °C targeted

P.lab             = 1                             # laboratory pressure in
atm
P.headspace       = P.lab * V.vial/(V.vial-V.aq)  # headspace pressure in a
tm

P.x               = 0.999  / 10^6  * P.headspace  # 0.999 is example N2O co
ncentration in ppm (micromol/mol);P.x partial gas pressure in atm mol mol-
1

# ######################


C.h      = (P.x  * V.hs)/(R * T)          # moles N2O in headspace

C.aq     =  P.x  * V.aq * K               # moles N2O in solution

C.total  =  C.h + C.aq; C.total           # total moles N2O of sample

n2o.aq.mol = C.total/V.aq               # total moles of N2O moles per uni
t water (unit water equals amount water injected to vial) # moles N2O / L

n2o.aq.mg  = C.total/V.aq * 44 * 1000   # convert moles N2O / L to mg N2O
/ L using molecular weight of N2O (44 g/mol)

n2o.aq.mg * (28/44) * 1000              # convert to microgram N-N2O / L
```

[1] Dong *et al.* 2006. Sources of nitrogen used for denitrification and nitrous oxide formation in sediments of the hypernutrified Colne, the nitrified Humber, and the oligotrophic Conwy estuaries, United Kingdom. *Limnol. Oceonagr.* 51 (1, part 2): 545-557. remark, SIF UC Davis recommends $ZnCl_2$ 50% w/v solution at 10μL/mL sample.

[2] Roper *et al.* 2013. A simple method for quantifying dissolved nitrous oxide in tile drainage water. *Can. J. Soil. Sci.* 93: 59-64
